# Ultrasonographically-guided fine-needle aspiration of axillary lymph nodes: role in breast cancer management

**DOI:** 10.1038/sj.bjc.6600744

**Published:** 2003-03-04

**Authors:** A Sapino, P Cassoni, E Zanon, F Fraire, S Croce, C Coluccia, M Donadio, G Bussolati

**Affiliations:** 1 Department of Biomedical Sciences and Human Oncology, University of Turin, 10126 Turin, Italy; 2 Department of Radiology, University of Turin, 10126 Turin, Italy; 3 Department of Oncology, San Giovanni Hospital, 10126 Turin, Italy

**Keywords:** lymph node, metastases, fine-needle aspiration, cytology, ultrasound, breast, carcinoma

## Abstract

The knowledge of the status of axillary lymph nodes (LN) of patients with breast cancer is a fundamental prerequisite in the therapeutic decision. In the present work, we evaluated the impact of fine-needle aspiration cytology (FNAC) of ultrasonographically (US) selected axillary LN in the diagnosis of LN metastases and subsequently in the treatment of patients with breast cancer. Axillary US was performed in 298 patients with diagnosed breast cancer (267 invasive carcinomas and 31 ductal carcinoma *in situ* DCIS), and in 95 patients it was followed by FNAC of US suspicious LN. Smears were examined by routine cytological staining. Cases of uncertain diagnosis were stained in immunocytochemistry (ICC) with a combination of anticytokeratin and anti-HMFG2 antibodies. Eighty-five FNAC were informative (49 LN were positive for metastases, 36 were negative). In 49 of 267 patients with invasive breast carcinoma (18%), a preoperative diagnosis of metastatic LN in the axilla could be confirmed. These patients could proceed directly to axillary dissection. In addition, US-guided FNAC presurgically scored 49 out of 88 (55%) metastatic LN. Of all others, with nonsuspicious LN on US (203 cases including 31 DCIS), in which no FNAC examination was performed, 28 invasive carcinomas (16%) turned out to be LN positive on histological examination. Based on these data, US examination should be performed in all patients with breast cancer adding ICC-supported FNAC only on US-suspect LN. This presurgical protocol is reliable for screening patients with LN metastases that should proceed directly to axillary dissection or adjuvant chemotherapy, thus avoiding sentinel lymph node biopsy.

Management of breast cancer patients is much improved since the introduction of multidisciplinary treatment centres. The relation between diagnosis and therapy is gradually becoming closer as ever more sophisticated diagnostic tools and new therapeutic strategies become available (Greco *et al*, 1998; Cady, 2000). For example, neoadjuvant chemotherapy can dramatically downstage both the primary tumour and the axilla, making many patients good candidates for breast-conserving surgical techniques (Singletary, 2001). However, at the same time, this poses new questions, such as the adequacy of diagnostic preoperative staging of the axillary lymph nodes (LN) in order to adopt specific therapeutic decisions. Besides clinical examination, different sophisticated imaging techniques have been proposed as alternative methods in the staging of axillary LN in breast cancer (Kvistad *et al*, 2000; Shimizu *et al*, 2000; Nishiyama *et al*, 2001). However, none of these procedures has been successful enough to replace histological examination. The introduction of the sentinel lymph node biopsy (SLNB) has partly solved the problem. In fact, the low rate of skip metastases in breast cancer gives to the SLN the role of reliable indicator of the status of the axilla, thereby avoiding nodal dissection, a surgical procedure that exposes patients to morbidity not justified by the benefit/risk ratio (Gervasoni *et al*, 2000). However, the main objections to SLNB are that the technique *per se* is quite complex and that the adequacy of the biopsy relies too much on the skill and experience of the surgeon (ADASP, 2001). In addition, the method used to perform histological examination of the SLN is debated and varies depending on the guidelines followed by the pathologist (ADASP, 2001; Kane *et al*, 2001; Turner *et al*, 2001; Veronesi *et al*, 2001).

To take the problem a step further, the ideal approach would be to employ a simple preoperative procedure that would allow selection of those patients who should directly undergo axillary dissection, sparing them SLNB. There are very few reports of image-guided LN fine-needle aspiration cytology (FNAC) as a means of improving presurgical staging of the axilla in breast cancer patients (Bonnema *et al*, 1997; Verbanck *et al*, 1997; de Kanter *et al*, 1999).

The aim of the present study was to evaluate the possibility of introducing ultrasonographic (US) examination of axillary LN in association with FNAC as a useful diagnostic tool in the presurgical management of patients with breast cancer.

## MATERIALS AND METHODS

### Case series

In 2000, a total of 298 patients with breast carcinomas were operated in the Breast Unit of the San Giovanni Hospital (Torino, Italy). All these patients underwent presurgical FNAC or core biopsy of the breast lesions. In both procedures, sample adequacy was immediately evaluated by rapid haematoxylin–eosin (H&E) staining of one smear. It was also possible to assess the adequacy of core biopsies by smearing the tissue fragments on a slide that was then fixed in methanol and immediately stained. Using this method, the number of core samples per case ranged from 1 to 3.

In addition, before surgery all these 298 patients underwent clinical and US examination of the ipsilateral axillary LN carried out by the radiologist using a linear array transducer, small parts probe 10 MHz, Hitachi ‘Astro’ (Hitachi Medical Systems, Tarrytown, BY, USA). The US features considered as suspect were two-dimensional enlargement giving a rounded appearance to the LN, echopoor central hilus and eccentricity of the nodal cortex. Ninety-five patients with LN presenting at least one of the previous features were considered suspect for metastases and therefore investigated by US-guided FNAC. When more than one LN suspected for metastasis was detected, only the largest or more suspicious one was aspirated, but when this turned out to be negative, the aspiration was performed on the other US-suspect LN. Several free-hand passes were made into each suspect LN using a 22-gauge needle. The number of US-recognised axillary LN was recorded and compared with the number of LN at axillary dissection, when performed.

### Smear preparation

The lymph node aspirated material was completely smeared onto two to three slides, immediately fixed in methanol and stained with rapid H&E. In order to recognise sporadic neoplastic cells, undetectable by routine staining, selected smears that were negative for cancer cells by H&E were evaluated by immuno-cytochemistry (ICC) with a locally produced cocktail (1 : 1 solution) of mouse monoclonal antibodies against two epithelial antigens, anticytokeratin (clone kl1, Immunotech, Marseille, France; dil. 1 : 50) and antiepithelial membrane antigen (clone HMFG-2, kindly supplied by J. Taylor Papadimitriou, London, UK; dil. 1 : 30). In brief, the slides were demounted, rehydrated in distilled H_2_O, rinsed in PBS and incubated for 30 min at room temperature with the cocktail of primary antibodies. The slides were then incubated for 20 min with a secondary biotinylated antibody and with streptavidin–peroxidase conjugate (1 : 50, StrAviGen MultiLink® Kit, BioGenex, S.Ramon, CA, USA) for another 20 min at room temperature. Finally, 3′-3-diaminobenzidine (DAB) chromogen solution (LiquidDAB Substrate Pack, BioGenex) was used to reveal the reactions.

The cytological diagnoses of the aspirated LN were compared with the histological diagnosis either of axillary LN dissection or of SLNB. Sensitivity, specificity and positive and negative predictive values of US-guided FNAC were calculated.

### Sentinel lymphnode examination

Sentinel lymph node were longitudinally bisected through the hilus, with further sectioning if the halves were thicker than 2 mm (Turner *et al*, 2001). The microscopic examination was assessed first on four sections stained with H&E and then negative LN were evaluated at two additional levels with ICC using the same cocktail of antibodies used for cytology.

## RESULTS

**Figure 1:**
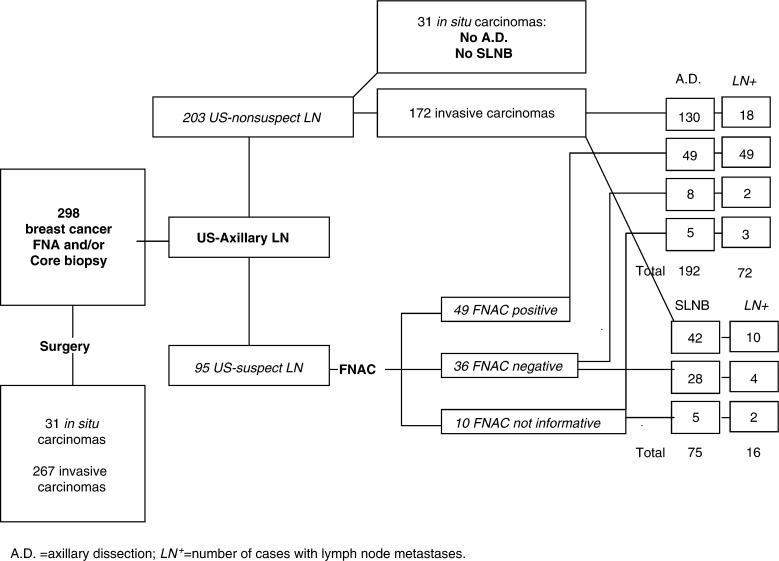
Diagnostic protocol of 298 patients with breast cancer.

Of the 298 breast cancer operated, 31 (10.5%) were *in situ* and 267 (89.5%) were invasive carcinomas. In 75 patients with invasive carcinomas, the SLNB was feasible and successfully performed; the other patients underwent axillary dissection. Lymph nodes metastases were present in 88 cases (33%) (pN+). Only 16 out of 75 (21.3%) SLN were affected by metastases (Figure 1). All patients underwent US examination before surgery. The number of US-recognised LN was in general lower than the number of LN at axillary dissection.

In 95 patients US of axillary LN was followed by FNAC. No clinical complications associated to FNAC were observed in any patient. Forty-nine samples were positive and 36 were negative for carcinoma cells and 10 samples were noninformative for diagnosis (Figure 1

and Table 1

**Table 1:** Correlation of tumour size with histological and FNAC diagnosis of axillary lymph node status in 95 patients

**Tumour size (cm)**	**FNAC– N-**	**FNAC– N+**	**FNAC+ N+**	**FNAC na N-**	**FNAC na N+**	**Total**
<0.5	4	0	0	0	0	4
0.5–1	13	0	2	0	3	18
1–2	8	4	18	5	1	36
>2	5	2	29	0	1	37

**Figure 2:**
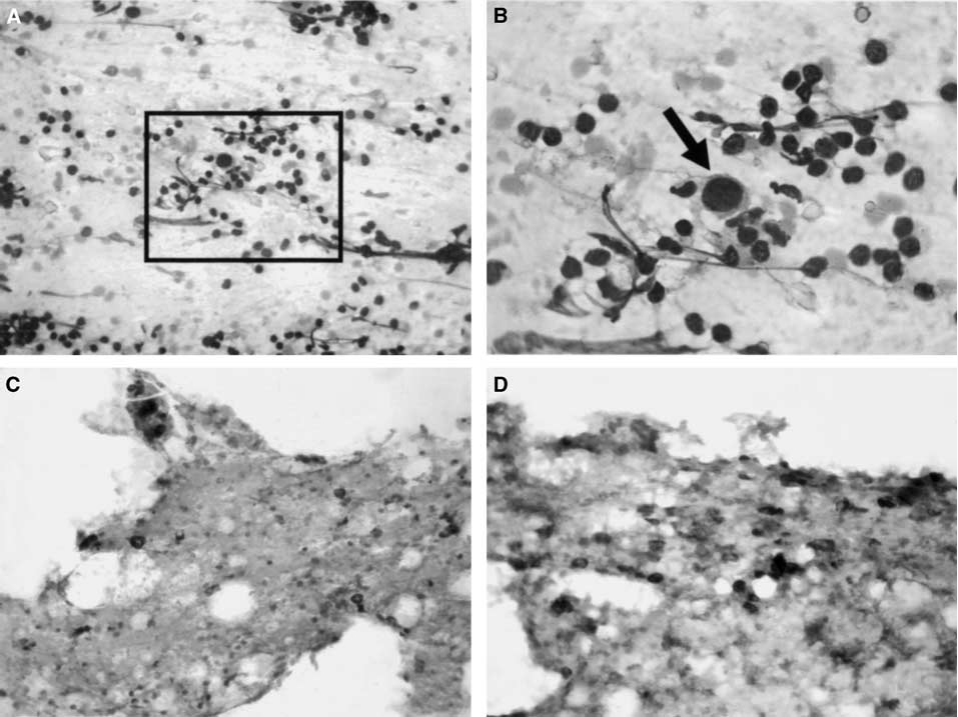
(**A**) Hematoxylin & Eosin stained smear of a US-guided FNAC in a selected axillary lymph node in breast cancer (×200 original magnification) (inset). (**B**) In a background of lymphocytes and red cells one large cell with hyperchromatic nucleus is evident (1000 × original magnification. (**C**), (**D**) The cocktail of antipancytokeratin and antiepithelial membrane antigen antibodies outlines numerous noncohesive metastatic cells of a lobular infiltrating carcinomas scattered among the lymphocytes (× 200 original magnification).

FNAC−=cytologically negative, FNAC+=cytologically positive, FNAC na=not adequate; N−=histologically negative, N+=histologically positive

). Comparison of the cytological results with the histological diagnosis provided by axillary dissection or by SLNB showed that FNAC was endowed with high sensitivity and absolute specificity as all 49 positive cases were true positives. In nine of these cases the cytological diagnosis of metastases was supported by cytokeratin and HMFG-2 immunostaining that outlined scattered cancer cells (Figure 2C, D

) (Table 2

**Table 2:** Impact of ICC on FNAC diagnosis of LN metastases

	**FNAC positive (49 cases)**		**FNAC negative (36 cases)**		**FNAC not adequate (10 cases)**	
**LIMPH node histology**	**ICC**	**H&E**	**ICC**	**H&E**	**H&E**	**Total**
No metastases	0	0	21	9	5	35
Metastases of 0.2–1 cm	8	33	6	0	3	52
Metastases of 1–2 cm	1	7	0	0	0	8

*Predictive values of FNAC*: Sensitivity=89%; specificity=100%; positive predictive value=100%; negative predictive value=82.3%.

). The immunostaining resulted particularly useful in seven cases where the primary tumour was an invasive lobular carcinoma. In nine patients selected for adjuvant chemotherapy, US-guided FNAC revealed the presence of LN metastases. Six of the 36 (16%) negative cytological diagnoses were false negatives since LN metastases were found by histology (Figure 1 and Table 2). In one case, the lymph node was affected by a micrometastasis (diameter p2 mm), in two cases, the lymph node metastasis was smaller than 4 mm and in three cases the metastases were smaller than 0.9 cm but invaded the LN capsule. In these false negative cases, ICC was also negative, suggesting that the FNAC sample was probably obtained either in a nonmetastatic LN area or in a reactive LN. Overall, the results indicate that ICC increases the sensitivity of routine cytological diagnosis (Table 2).

In 10 cases, the cellularity of the FNAC samples was too poor to consent diagnosis (Figure 1). Despite this, the FNAC procedure was not repeated because the position of the LN was too difficult to be reached or the node was too small. In five of these cases, histology showed the presence of metastatic LN, none larger than 5 mm (Table 2).

## DISCUSSION

In the present study, we demonstrate that US-guided FNAC of axillary LN in breast cancer patients can reliably predict the presence of metastases and therefore refer the patients to the appropriate surgical treatment, avoiding the SLNB. In fact, in our series, US-guided FNAC presurgically screened 18% of invasive breast carcinoma operated in the same year and scored 49 out of 88 (55%) of the metastatic LN.

Since the early 1980s, US examination has been proposed by many authors as one of the most successful procedures for evaluating the status of the axilla in breast cancer patients before surgery (Bruneton *et al*, 1986; Pamilo *et al*, 1989; Mustonen *et al*, 1990; Yang *et al*, 1996; Strauss *et al*, 1998;). Sonography can easily explore the different nodal chains and, when LN are found, specific signs may be searched to evaluate the presence of metastases (Feu *et al*, 1997; Rizzatto, 2001). We here reported a 16% false negative rate by US alone, since 28 out of 172 of US-nonsuspect LN were positive at histology. However, a great improvement of the diagnostic accuracy emerged when US examination was combined with FNAC of LN suspect to be metastatic, as demonstrated by the few available reports on the subject (Bonnema *et al*, 1997; Verbanck *et al*, 1997; de Kanter *et al*, 1999,^2000^). In fact, in our study and in previous ones that used a similar approach, the cytological diagnosis of metastases reached 100% specificity. On the other hand, Bonnema *et al* (1997) reported 80% sensitivity and a 76% negative predictive value of the cytological method. In the present work, we observed an increase in both sensitivity (89%) and negative predictive value (84%), which has to be related to the additional use of ICC. The use of a cocktail of antiepithelial membrane antigen and antipancytokeratin antibodies for identifying scattered epithelial cancer cells has been proposed recently in bone marrow smears (Gebauer *et al*, 2001). The possibility of using the same smears obtained for routine H&E staining for additional ICC analyses with the cocktail of antibodies recognising different antigens is an interesting approach, which reduces further sampling of the LN, and limits potential diagnostic pitfalls. In fact, cytokeratin-positive interstitial reticulum cells present in normal or reactive LN may complicate interpretation of the results (Linden and Zarbo, 2001) and, on the other hand, the epithelial membrane antigen may enhance both sensitivity and specificity of the procedure, particularly for metastases of invasive lobular breast cancer. In the present series, in seven out of nine metastatic cases diagnosed by ICC, the primary tumour was an invasive lobular carcinoma with scattered neoplastic cells dispersed among lymphocytes and clearly outlined only by ICC.

In a recent multicentre study, de Kanter *et al* (1999) demonstrated that, using US combined with FNAC in patients without palpable axillary nodes, SLNB could be avoided in 17% of cases since cytology had already diagnosed axillary metastases. Similarly, in our experience, 18% of metastatic invasive carcinomas were already diagnosed using US-guided FNAC, which presurgically scored 49 out of 88 (55%) of the metastatic LN.

In addition, the method could be particularly valuable in cases of extensive metastatic involvement, which is the cause of false negative SLNB procedures (Dequanter *et al*, 2001). In fact, when a LN is completely replaced by the tumour, there is poor uptake of radioactivity. In our series, the whole group of pN2 LN were positive by cytological examination.

The requirement of neoadjuvant chemotherapy appears to be an additional indication for US-guided FNAC LN examination. Systemic chemotherapy has been broadened to include all invasive tumours larger than 1 cm regardless of axillary status (Fisher *et al*, 1998). Nevertheless, to establish the metastatic status of LN can be useful for evaluating the response to chemotherapy and the prognostic work-up of patients who are not surgical candidates. In the present series, nine of the patients enrolled by the oncologist for neoadjuvant chemotherapy were diagnosed with LN metastases using the guided FNAC procedure.

Finally, in a quality control programme, the evaluation of the standard goal that range from 32 to 40% of metastases in the SLNB (de Kanter *et al*, 2000; Rahusen *et al*, 2001; Veronesi *et al*, 2001) has to be re-evaluated taking into account that most of the metastatic LN can be already diagnosed with US-guided FNAC. In the present series, the selection of cases markedly reduces the percentage of metastatic SLNB with respect to the values accepted in the literature and, at the same time, increases the number of axillary dissection as first-choice treatment. Thus, US-guided FNAC of axillary LN could generate cost savings to the health care system by reducing the added cost incurred by subsequent axillary dissection for the patients who show metastatic SLN.

In conclusion, because of its low cost and high specificity, we pro-pose that examination by US of the axilla in patients with diagnosed breast cancer should be performed any time before surgery. FNAC of selected LN should be added to the diagnostic protocol, in order to directly schedule patients with a cytological diagnosis of LN metastases to the appropriate treatment, avoiding the SLNB.
